# Risk Assessment on Suicide Death and Attempt among Chinese Rural Youths Aged 15–34 Years

**DOI:** 10.3390/ijerph182413362

**Published:** 2021-12-18

**Authors:** Long Sun, Jie Zhang, Dorian A. Lamis, Yifan Wang

**Affiliations:** 1 Center for Suicide Prevention Research, School of Public Health, Shandong University, 44 Wenhuaxi Road, Jinan 250012, China; sunlong@sdu.edu.cn (L.S.); yifanw@mail.sdu.edu.cn (Y.W.); 2 Key Laboratory for Health Economics and Policy Research (Shandong University), National Health Commission of China, 44 Wenhuaxi Road, Jinan 250012, China; 3 Department of Sociology, Central University of Finance and Economics, 39 Xueyuannan Road, Beijing 100081, China; 4 Department of Sociology, State University of New York Buffalo State, 1300 Elmwood Avenue, Buffalo, NY 14222, USA; 5 Department of Psychiatry and Behavioral Sciences, Emory University School of Medicine, 10 Park Place, Atlanta, GA 30303, USA; dorian.lamis@emory.edu

**Keywords:** suicide attempt, suicide death, risk assessment, rural China

## Abstract

**Background:** Although many suicide risk assessment tools are available in the world, their validity is not adequately assessed. In this study, we aimed to develop and evaluate a suicide risk assessment model among Chinese rural youths aged 15–34 years. **Method:** Subjects were 373 suicide deaths and 507 suicide attempters aged 15–34 years in three Chinese provinces (Shandong, Liaoning, and Hunan). Information about the community residents was also collected as the control groups. Social-demographic, social and psychological variables were examined for the suicides, suicide attempters, and community residents. Logistic regressions based on subjects from Shandong and Liaoning provinces were conducted to establish the suicide risk assessment models. Receiver operating characteristic (ROC) curves were drawn, and area under the ROC curves (AUC) were calculated to show how well the models separated the group being tested into those with and without suicide attempt or suicide. **Results:** The assessment model for suicide death included education years (OR = 0.773, *p* < 0.001), agricultural worker (OR = 2.091, *p* < 0.05), physical health (OR = 0.445, *p* < 0.05), family suicide history (OR = 6.858, *p* < 0.001), negative life events (OR = 1.340, *p* < 0.001), hopelessness (OR = 1.171, *p* < 0.001), impulsivity (OR = 1.151, *p* < 0.001), and mental disorder (OR = 8.384, *p* < 0.001). All these factors were also supported in the assessment model for suicide attempt, with an extension of very poor economic status (OR = 1.941, *p* < 0.01) and social interaction (OR = 0.855, *p* < 0.001). The AUC was 0.950 and 0.857 for the sample used to establish the assessment models of suicide death and attempt, respectively. The AUC was 0.967 and 0.942 for the sample used to verify the established assessment models of suicide death and attempt, respectively. **Conclusions:** Compared with some other assessment tools, the models for suicide death and attempt in the current study performed well among Chinese rural youths aged 15–34 years. A reliable suicide risk assessment approach, which includes multiple risk factors, should be evaluated in various cultures and populations.

## 1. Introduction

The World Health Organization (WHO) reported that there were an estimated 700,000 suicide deaths worldwide in 2019 [1], and the number of suicide attempts has been estimated to be 20 times higher than suicide deaths [2]. China had one of the highest suicide rates in the 1990s in the world [3]. Although Chinese suicide rates have decreased in recent decades, suicide prevention continues to be of crucial importance, especially in rural areas [4]. The proportion and rank of suicide deaths also varied greatly by age in different regions. Globally, suicide accounted for 8.5% of all deaths and was ranked as the second leading cause of death among young adults aged 15–29 years [5]. In China, rural suicides aged 15–34 years were at higher risk of suicide, which had been reported in previous studies [6]. Thus, we should prioritize addressing suicide among Chinese rural young adults.

In recent decades, efforts have promoted the development of suicide prevention strategies, and a wide spectrum of risk factors has been recognized for suicide behaviors. It had been established that suicidal behavior was caused by social, psychological, cultural, and other factors [5]. In the process of suicide assessment and intervention, a key component of prevention strategies must include the identification of high-risk individuals [7].

There were many approaches used to assess suicide risk. In the 1990s, these tools included two kinds of methods. The first method of suicide risk assessment was based on standardized scales assessing ideation and behaviors, such as the Suicide Intent Scale [8] and the Scale for Suicide Ideation [9]. Previous studies had reported their value in predicting suicide [10]. However, the validity was not at a high level [11,12]. The second method consisted of psychological scales about the risk factors of suicide, such as the Beck Depression Inventory (BDI) [13], Beck Hopeless Scale (BHS) [14], dexamethasone suppression test (DST) [15], and cerebrospinal fluid (CSF) 5-hydroxyindoleacetic acid (5-HIAA) concentration test [16]. Given the many various risk factors to be considered, the validity of assessing suicide risk was also limited [17].

In recent years, we have recognized that comprehensive suicide risk assessment should be undertaken to ensure the accuracy of the assessment. The WHO’s mhGAP Intervention Guide recommended assessing any person who experienced a medically serious act of self-harm, chronic pain, or severity of emotional symptoms [18]. The Columbia-Suicide Severity Rating Scale (C-SSRS) is another tool, often considered the gold standard of suicide assessment, which had been translated into several languages. The definitions of ideation and behavior incorporated in the C-SSRS have also been used by the US Food and Drug Administration to classify the potentially suicidal adverse events [19], which essentially asked people about their suicidal thoughts and past suicidal behaviors. There are also other suicide risk assessment tools, such as the SAD PERSONS scale [20], Suicide Status Form (SSF) [21], and Collaborative Assessment and Management of Suicidality (CAMS) [22]. In China, there are also some risk assessment tools, which have been used and evaluated among some special populations. The Nurses’ Global Assessment of Suicide Risk scale (NGASR) has been translated and applied to patients with mental disorders [23]. The C-SSRS has been translated and applied to middle school students [24]. There have been some studies that have built risk assessment tools among inpatients [25] and college students [26].

Although the sensitivity and specificity of these comprehensive tools had been demonstrated in previous studies [27,28,29], there were also limitations that should be further explored. First, we have identified many factors associated with suicidal behavior, but most of these tools only considered a handful of these factors. Second, the effects of these risk factors varied with regard to suicide behavior, and most of these tools did not consider the effect size of different factors. Third, most of these tools were developed in Western countries, particularly in the United States, so they might not be suitable for individuals residing in other countries such as China [30]. Thus, further works need to be completed to strengthen the accuracy of suicide risk assessment in China.

In the current study, our aims were to (1) select the important factors associated with suicide attempts and deaths among Chinese rural young adults, (2) identify the effect size numerical relationship of these risk factors for suicide attempt and death, and (3) evaluate the performance of the risk assessment models among Chinese rural young adults. Findings from this study may be helpful for suicide prevention in China as well as elsewhere in the world.

## 2. Materials and Methods

### 2.1. Study Sample and the General Design

We collected data from two samples of suicide deaths and attempts in the same three China provinces, which included Shandong, Liaoning, and Hunan. All of these three provinces represented a higher agriculture development size in China [31]. We selected 16 rural counties in all of the provinces to collect the data about suicide deaths between October 2005 and June 2008, and 13 rural counties were selected to collect the data about suicide attempts between June 2012 and June 2015.

In each rural county, suicide deaths and attempts among individuals aged 15–34 years were consecutively recruited. For suicides, the county-level Center for Disease Control and Prevention (CDC) monitored suicide occurrences and informed the research group each month. The interview was scheduled between 1 and 2 months after suicide deaths. We employed the psychological autopsy (PA) method to collect data about suicides, which had been verified in a previous study in China [32]. For each suicide, there were at least two informants who were interviewed. The guidelines for inclusion of informants and decision of their biased reports had been reported in a former study [33]. The suicide attempters were collected from the hospital emergency departments in these rural counties. The hospital emergency departments would notify the research team of any suicide attempters on a monthly basis. The research team should verify the recruitment process and schedule the interview between 1 and 3 months after the suicide incidences.

There were also strict inclusion criteria for the suicides, suicide attempters, and community residents. The inclusion criteria were (1) the attempters whose injury and wounds were so serious as to require hospitalization or immediate medical care, (2) aged 15–34 years, and (3) living in a rural region for more than 6 months. Moreover, the community residents were systematically and randomly selected from the same or neighboring rural village with the suicides and suicide attempters, and approximately matching the gender and age distribution (discrepancy less than 3 years). Prior to the interview, every interviewer received comprehensive training from the psychiatrists and the study designers. All the interviewers were master students who had majored in public health or psychiatry. To ensure data quality, there were also supervisors who checked the completed questionnaires in the evening.

### 2.2. Interview Procedure

Prior to the onset of the interview, the local health agency or the village administration visited the participants in order to increase the participation rate. Then, the interviewer would give an overview of this study and introduce the harms and benefits of participation. Once consent was obtained, an appointment was scheduled for a face-to-face interview. The interview took place privately in a private place of a village medical room or their home. However, among the attempters who were too weak to talk for a long time, their family members were able to assist them by answering some questions in the protocol. In total, we found 416 suicides and 578 suicide attempters in this study. Among these subjects, 392 suicides and 523 suicide attempters participated in this study. Because of the missing data problem, we finally analyzed 373 suicides and 507 suicide attempters to build and verify the assessment models of suicide and suicide attempt. The valid response rate for suicides was 89.7% (373/416), and the valid response rate for suicide attempts was 87.7% (507/578). The average time for each interview was approximately 1.5 h.

### 2.3. Measures

In an attempt to assess the risk of suicide death and attempt, we evaluated many factors that have been identified in previous studies among Chinese rural youth, such as gender, age, education, marital status, economic status, living alone, religious belief, pesticide at home, family suicide history, negative life events, hopelessness, social support, impulsivity, and mental disorder [33,34,35,36,37]. In the current study, all of these factors were used to build the risk assessment models of suicide and suicide attempt.

Gender was investigated and coded by male (1) and female (0). Age was measured by the subjects’ date of birth. The age for the suicides and suicide attempters was calculated from the time the suicidal behavior occurred, while the age for the community residents was calculated from the time when they were interviewed. Education years were evaluated by the number of years, which the subjects were educated in the school. Marital status was assessed as single, married, divorced, separated, or widowed. As few subjects were divorced, separated, or widowed, we recoded the response options of married status into never married (0) and ever married (1), which the latter one included married, divorced, separated, and widowed. Economic status was measured by the position of economic status in their village and response options, including very rich, rich, average, poor, and very poor. Consistent with previous studies [38], the dichotomous variable used was very poor (1) and others (0). Living alone was assessed by yes (1) and no (0). Occupation was assessed by agricultural worker, businessman, public service staff, student, factory worker, rural doctor, teacher, housewife, unemployed, and other. Given the high percentage of agricultural workers, it was recoded into agricultural worker (1) and others (0). 

Religious belief was measured by which religion the subjects believed in. The answers were no belief, Taoism, Muslim, Christianity, Buddhism, and others. As most of the subjects did not have a religious belief, we recoded into yes (1) and no (0). As the high percentage of suicide by pesticide [39], pesticide at home was assessed by a question regarding available pesticide at their home. The response options included yes (1) and no (0). Family suicide history was evaluated by one question asking if their family members ever experienced suicide behaviors (yes = 1; no = 0). 

A revised Chinese version of the Interview for Recent Life Events (IRLE) was used to measure negative life events, which the participants experienced during the past 12 months. The original version of IRLE included 64 items [40], and the research team added 19 specific events referring to Chinese culture. The revised Chinese version has been evaluated in a previous study [41]. For each event, the participants were also asked to distinguish whether it was a positive or negative experience. In the current study, only the number of negative life events was included in the analyses.

Hopelessness was assessed by the Chinese version of the Beck Hopelessness Scale (BHS) [14]. This scale consists of 20 items measured on a Likert scale from 1 (strongly disagree) to 5 (strongly agree). Previous studies have evaluated the Chinese version of the BHS and identified that it was a good scale with solid reliability and validity among Chinese adolescents [42].

The Chinese version of the Duke Social Support Index (DSSI) was used to measure the level of social support [43]. It contains three subscales (social interaction, subjective support, and instrumental support). The social interaction subscale includes four items, with response opinions ranging from 1 (nobody), 2 (1–4 people), or 3 (5 or more people). The subjected support subscale contains seven items, and each of them contains three response options from 1 (never) to 3 (frequently). There are 12 items in the instrumental support subscale with two response options, including yes (1) and no (0). The Chinese version of DSSI has demonstrated good reliability and validity among Chinese rural young populations [44].

Impulsivity was measured by a Chinese version of the Dickman Impulsivity Inventory (DII) [45]. There are 23 items in this scale to evaluate impulsiveness in participants’ daily lives. Response options were yes (1) or no (0) for each item. Previous studies have shown that the Chinese version of DII had good validity among Chinese rural youths [46].

The Chinese version of the Structured Clinical Interview for DSM-IV Axis I disorder (SCID) was used to diagnose mental disorders among the subjects [47]. The SCID has been successfully used for the diagnosis of mental disorders in mainland China, Hong Kong, Macau, and Taiwan [48]. This tool can identify 27 Axis I diagnoses. In the current study, the interviewers were asked to write information on the SCID book, and psychiatrists were recruited to make the final diagnoses of mental disorders for all of the suicide, suicide attempters, and community residents. Given the low percentages for each disorder among community residents, the dichotomous variable (yes/no) was analyzed in this study.

### 2.4. Ethics Statement

The study protocol and the ethical methodology were approved by the institutional review board (IRB) from the Chinese institutions (Shandong University, Central South University, and Dalian Medical University) and the University of New York, Buffalo State. We also followed the rules about human subject protection regulated by the NIMH, which funded the project. Informed consent was obtained from all participants in the study. For participants aged under 18 years, informed consent was obtained from their parents or legal guardian.

### 2.5. Statistical Methods

SPSS 24.0 for Windows (IBM, Armonk, NY, USA) was used for all data analyses in this study. We used *t*-tests or chi-square tests to compare the significance between suicide, suicide attempters, and community residents. Backward logistic regression analysis was performed to test the prediction model for suicide deaths and attempts. Following the construction of the risk factor model, the data in Hunan were used to validate it. All tests were two-tailed, and a *p* value of ≤ 0.05 was considered statistically significant. Receiver operating characteristic (ROC) curves were drawn, and area under the ROC curves (AUC) were used to test the validity of the model, which is an index to show how well the models separate the group being tested into those with and without suicide attempt and suicide. A rough guide for AUC scores about accuracy of classifying for the diagnostic models is that 0.90–1.00 = excellent; 0.80–0.90 = good; 0.70–0.80 = fair; 0.60–0.70 = poor; 0.50–0.60 = fail.

## 3. Results

In the current study, the participants in Shandong and Liaoning Province were selected to build the assessment models of suicide attempt and suicide. In addition, we used the participants in Hunan Province to verify the validity of the models.

### 3.1. Study Samples

In Table 1, we describe the data and compare the variables between suicides, suicide attempters, and community residents among training samples. When comparing suicide deaths with community residents, significant risk factors included age (26.18 vs. 25.09, *p* < 0.05), fewer education years (7.16 vs. 8.67, *p* < 0.001), very poor economic status (49.4% vs. 15.3%, *p* < 0.001), religious belief (28.3% vs. 15.3%, *p* < 0.001), physical health (32.1% vs. 14.5%, *p* < 0.001), pesticide at home (74.7% vs. 63.3%, *p* < 0.01), family suicide history (22.6% vs. 3.3%, *p* < 0.001), experience of greater negative life events (3.48 vs. 1.44, *p* < 0.001), hopelessness (69.77 vs. 47.85, *p* < 0.001), lack of social interaction (6.14 vs. 7.40, *p* < 0.001), lack of subjected social support (14.48 vs. 18.43, *p* < 0.001), lack of instrumental social support (9.11 vs. 10.64, *p* < 0.001), impulsivity (14.53 vs. 12.21, *p* < 0.001), and diagnosis of mental disorder (45.3% vs. 4.4%, *p* < 0.001). When comparing suicide attempters to community residents, the significant risk factors were the same as those in suicide deaths, with the exception of age (26.48 vs. 26.56, *p* > 0.05) and pesticide at home (61.9% vs. 58.8%, *p* > 0.05).

### 3.2. Suicide Risk Assessment Model

In this study, multivariate logistic regressions (backward method) were used to test the factors (see Table 2). We also reported the β values in the two models. Results indicate that education years (OR = 0.773, *p* < 0.001), agricultural worker (OR = 2.091, *p* < 0.05), physical health (OR = 0.445, *p* < 0.05), family suicide history (OR = 6.858, *p* < 0.001), negative life events (OR = 1.340, *p* < 0.001), hopelessness (OR = 1.171, *p* < 0.001), impulsivity (OR = 1.151, *p* < 0.001), and mental disorder (OR = 8.384, *p* < 0.001) were associated with suicide death. With regards to suicide attempts, significant factors were education years (OR = 0.811, *p* < 0.001), very poor economic status (OR = 1.941, *p* < 0.01), agricultural worker (OR = 1.847, *p* < 0.001), family suicide history (OR = 4.182, *p* < 0.01), negative life events (OR = 1.622, *p* < 0.001), hopelessness (OR = 1.063, *p* < 0.001), social interaction (OR = 0.855, *p* < 0.001), impulsivity (OR = 1.067, *p* < 0.01), and mental disorder (OR = 2.325, *p* < 0.05).

### 3.3. Validity of The Assessment Model among Training Samples

**Figure 1:**
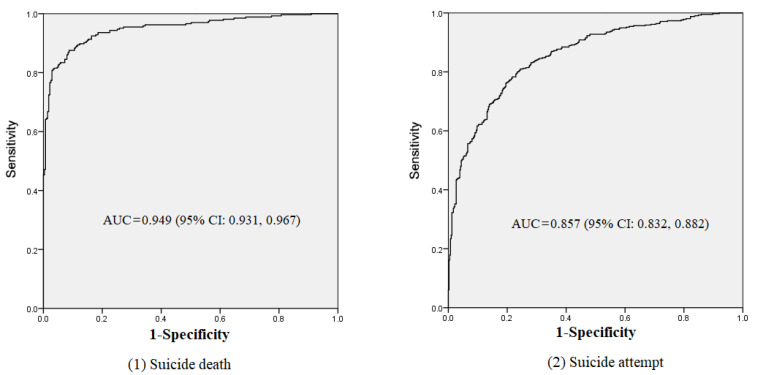
AUC for training sample.

A ROC curve and AUC were used to test the validity of the assessment model among training samples. The ROC curves are shown in Figure 1. In the suicide death sample, the AUC was 0.949 (95% CI: 0.931, 0.967), and the AUC was 0.857 (95% CI: 0.832, 0.882) for suicide attempt.

### 3.4. Performance of the Assessment Model

**Figure 2:**
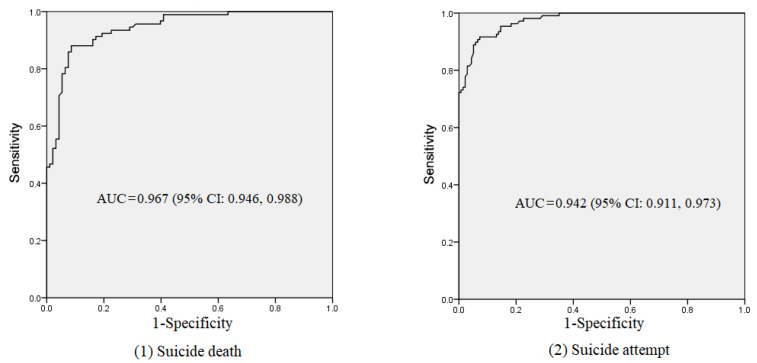
AUC for verification sample.

In order to assess the performance of these models, the characteristics of this sample in Hunan were described (Table 3). Then, the probability of suicide death and attempt for each participant in Hunan were calculated with the β values in Table 2. ROC curves and AUC were also used to verify the performance of the assessment models (Figure 2). In the verification sample of suicide deaths, AUC was 0.967 (95% CI: 0.946, 0.988), and AUC was 0.942 (95% CI: 0.911, 0.973) in the verification sample of suicide attempts.

## 4. Discussion

In China, many factors associated with suicidal behaviors have been identified, and using this data to inform practice and prevent suicide is necessary. One effective method is to assess suicide risk factors, which can aid in the early intervention of at-risk individuals. However, it is unreasonable to use all of these factors to assess suicide risk, and we should select the most useful ones for the population of interest. Thus, our first aim in this study was to select important risk factors, which were associated with suicide attempts and deaths in Chinese rural young adults.

In order to select pertinent suicide risk factors, we considered approximately 20 factors in this study. All of these factors were associated with suicidal behavior in rural China, which have been identified in previous studies [49,50]. The backward logistic regression approach demonstrated that suicide death was associated with education years, agricultural worker, physical health, family suicide history, negative life events, hopelessness, impulsivity, and mental disorder. All these factors were also supported in the assessment model for suicide attempt, with an extension of very poor economic status and social interaction. The selected risk factors for suicide deaths and attempts among Chinese rural young adults have been reported in previous studies [51,52]. When we examined these factors worldwide, we can also see that they are also strongly associated with suicidal behaviors in Western countries [53,54,55], which implies that all of these selected risk factors were important.

We also examined a risk assessment model to assess suicide risk. In Table 2, we reported the coefficients, OR, and associated 95% CI. To evaluate this model, we analyzed the AUC for the training sample. The AUC (0.949 for suicide death and 0.857 for suicide attempt) in Figure 1 further demonstrated that this was a good model to assess suicide risk among Chinese rural young adults. Previous studies reported the AUC was mainly about 0.80 [56,57,58], and the validities for the two models were higher than the validities in many previous studies. To further identify the performance of the risk assessment model, we tested it in a separate sample in south China. The results also indicated that it was a good model, which demonstrated high levels of AUCs for suicide risk assessment (0.967 and 0.942). Thus, we believe this is a good model to assess suicide risk and provides information about suicidal behavior among rural Chinese youth aged 15–34 years. The results may be explained by more factors that were considered in this study.

As we introduced before, suicide behavior is complicated and there are many associated factors that can promote it. In recent years, conflicting results about risk factors for suicide may exist due to cultural and societal differences between China and Western countries [59]. For example, religious belief is a protective factor for suicidal behavior in Western countries [60], but it is a risk factor in China [61]. This may be the reason why the performance of many risk assessment models was not good in China [62], and we need to build different models in different regions. In the current study, we built suicide risk assessment models under Chinese culture and society, and they may offer some implications for some other Asian countries with a similar culture.

In the current study, there were some limitations that should also be noted. First and foremost is the representativeness of the community residents group. Specifically, the sample size in the community residents is relatively small, and it may not represent the characteristics of the rural young adults aged 15–34 years. However, this is a more cost-effective method to assess suicide risk. Second, as previously discussed, this model may not be generalizable to other cultures or populations. However, we suggest evaluating risk assessment models across cultural samples. Finally, the design for the current study was not a cohort study, and the reliabilities are also at a lower level. However, it is a cost-effective method, even for suicide risk assessment.

## 5. Conclusions

Despite the aforementioned limitations, the present study addressed several gaps in the literature regarding suicide risk assessment. The majority of the related factors and the nice sensitivity of the models remind us that suicide risk assessment should comprehensively consider demographic, social, and psychological factors in clinical assessment and practices. In addition, our results need to be replicated in order to confirm the usefulness of the proposed suicide risk assessment model. Moreover, the findings contribute to our understanding of suicide risk and encourage the development and evaluation of assessment models incorporating multiple risk factors in different cultures and populations, which should inform suicide prevention efforts in China and throughout the world.

## Figures and Tables

**Table 1:** Description and single-factor analysis for suicide death and attempt among training sample (*n* (%) or mean ± SD).

	Suicide Death (*n* = 540)		$t/\chi^{2}$	Suicide Attempt (*n* = 825)		$t/\chi^{2}$
	Case	Control		Case	Control	
*n*	265 (49.1)	275 (50.9)		415 (50.3)	410 (49.7)	
Gender			0.93			0.01
Male	143 (54.0)	137 (49.8)		170 (41.0)	169 (41.2)	
Female	122 (46.0)	138 (50.2)		245 (59.0)	241 (58.8)	
Age	26.18 ± 6.17	25.09 ± 6.23	2.04 *	26.48 ± 5.28	26.56 ± 5.28	−0.24
Education years	7.16 ± 2.64	8.67 ± 2.11	−7.38 ***	7.47 ± 2.86	9.18 ± 2.93	−8.49 ***
Marital status			1.72			2.29
Never married	89 (33.6)	78 (28.4)		59 (14.2)	44 (10.7)	
Ever married	176 (66.4)	197 (71.6)		356 (85.8)	366 (89.3)	
Economic status			72.33 ***			36.43 ***
Very poor	131 (49.4)	42 (15.3)		113 (27.2)	44 (10.7)	
Others	134 (50.6)	233 (84.7)		302 (72.8)	366 (89.3)	
Living alone			2.98			0.70
Yes	21 (7.9)	12 (4.4)		13 (3.1)	9 (2.2)	
No	244 (92.1)	263 (95.6)		402 (96.9)	401 (97.8)	
Occupation			0.64			0.30
Agricultural worker	121 (45.7)	135 (49.1)		227 (54.7)	232 (56.6)	
Others	144 (54.3)	140 (50.9)		188 (45.3)	178 (43.4)	
Religious belief			13.50 ***			4.25 *
Yes	75 (28.3)	42 (15.3)		81 (19.5)	58 (14.1)	
No	190 (71.7)	233 (84.7)		334 (80.5)	352 (85.9)	
Pesticide at home			8.25 **			0.85
Yes	198 (74.7)	174 (63.3)		257 (61.9)	241 (58.8)	
No	67 (25.3)	101 (36.7)		158 (38.1)	169 (41.2)	
Family suicide history			45.43 ***			19.31 ***
Yes	60 (22.6)	9 (3.3)		35 (8.4)	7 (1.7)	
No	205 (77.4)	266 (96.7)		380 (91.6)	403 (98.3)	
Negative life events	3.48 ± 2.33	1.44 ± 1.57	11.97 ***	1.84 ± 0.66	0.66 ± 1.10	11.15 ***
Hopelessness	69.77 ± 13.79	47.85 ± 7.93	22.73 ***	51.04 ± 15.73	36.94 ± 10.79	15.00 ***
Social interaction	6.14 ± 1.73	7.40 ± 1.69	−8.56 ***	7.75 ± 2.25	9.08 ± 1.85	−9.20 ***
Subjected support	14.48 ± 3.40	18.43 ± 2.40	−15.65 ***	19.33 ± 33.33	20.10 ± 1.51	−0.47 *
Instrumental support	9.11±2.86	10.64±1.86	−7.37 ***	10.87±2.13	11.46±1.34	−4.81 ***
Impulsivity	14.53±5.24	12.21±3.76	5.91 ***	10.30±4.14	9.12±3.01	4.70 ***
Mental disorder			122.35 ***			56.81 ***
Yes	120 (45.3)	12 (4.4)		83 (20.0)	13 (3.2)	
No	145 (54.7)	263 (95.6)		332 (80.0)	397 (96.8)	

Note: * *p* ≤ 0.05; ** *p* ≤ 0.01; *** *p* ≤ 0.001.

**Table 2:** Backward logistic analysis for suicide death and attempt among training sample.

	Suicide Death (*n* = 540)				Suicide Attempt (*n* = 825)		
	β	OR	95% CI		β	OR	95% CI
Education years	−0.243	0.784 ***	0.685, 0.898		−0.209	0.811 ***	0.757, 0.869
Very poor	-	-	-		0.663	1.941 **	1.198, 3.144
Agricultural worker	0.772	2.163 *	1.175, 3.984		0.614	1.847 ***	1.271, 2.685
Family suicide history	1.919	6.817 ***	2.338, 19.875		1.431	4.182 **	1.463, 11.951
Negative life events	0.258	1.294 **	1.097, 1.525		0.484	1.622 ***	1.393, 1.888
Hopelessness	0.152	1.164 ***	1.127, 1.201		0.061	1.063 ***	1.046, 1.080
Social interaction	-	-	-		−0.157	0.855 ***	0.782, 0.934
Impulsivity	0.140	1.151 ***	1.078, 1.228		0.065	1.067 **	1.016, 1.121
Mental disorder	2.042	7.705 ***	3.081, 19.266		0.844	2.325 *	1.089, 4.963
Constant	−10.062	0.000 ***	-		−1.283	0.277	0.757, 0.869
R^2^	0.751				0.481		

Note: OR = odds ratio; CI = confidence interval; * *p* ≤ 0.05; ** *p* ≤ 0.01; *** *p* ≤ 0.001.

**Table 3:** Description and single-factor analysis for suicide death and attempt among verification sample (*n* (%) or mean ± SD).

	Suicide Death (*n* = 245)		$t/\chi^{2}$	Suicide Attempt (*n* = 185)		$t/\chi^{2}$
	Case	Control		Case	Control	
*n*	108 (44.1)	137 (55.9)		92 (49.7)	93 (50.3)	
Gender			1.16			0.01
Male	57 (52.8)	63 (46.0)		35 (38.0)	36 (38.7)	
Female	51 (47.2)	74 (54.0)		57 (62.0)	57 (61.3)	
Age	26.91 ± 5.96	26.60 ± 5.67	0.41	27.84 ± 5.33	27.72 ± 5.10	0.15
Education years	8.09 ± 3.05	10.12 ± 2.64	−5.56 ***	9.35 ± 2.58	12.40 ± 3.71	−6.49 ***
Marital status			1.00			2.42
Never married	33 (30.6)	34 (24.8)		16 (17.4)	25 (26.9)	
Ever married	75 (69.4)	103 (75.2)		76 (82.6)	68 (73.1)	
Economic status			48.08 ***			6.64 **
Very poor	53 (49.1)	13 (9.5)		16 (17.4)	5 (5.4)	
Others	55 (50.9)	124 (90.5)		76 (82.6)	88 (94.6)	
Living alone			6.24 *			2.55
Yes	13 (12.0)	5 (3.6)		7 (7.6)	14 (15.1)	
No	95 (88.0)	132 (96.4)		85 (92.4)	79 (84.9)	
Occupation			6.91 **			13.66 ***
Agricultural worker	66 (61.1)	105 (76.6)		60 (65.2)	82 (88.2)	
Others	42 (38.9)	32 (23.4)		32 (34.8)	11 (11.8)	
Religious belief			2.82			0.05
Yes	33 (30.6)	29 (21.2)		22 (23.9)	21 (22.6)	
No	75 (69.4)	108 (78.8)		70 (76.1)	72 (77.4)	
Pesticide at home			8.20 **			8.75 **
Yes	86 (79.6)	86 (62.8)		36 (39.1)	18 (19.4)	
No	22 (20.4)	51 (37.2)		56 (60.9)	75 (80.6)	
Family Suicide history			16.56 ***			4.77 *
Yes	23 (21.3)	6 (4.4)		7 (7.6)	1 (1.1)	
No	85 (78.7)	131 (95.6)		85 (92.4)	92 (98.9)	
Negative life events	2.99 ± 2.31	0.45 ± 0.95	11.65 ***	2.07 ± 2.18	0.24 ± 0.48	7.91 ***
Hopelessness	67.62 ± 12.49	44.97 ± 7.91	17.29 ***	60.39 ± 8.81	44.29 ± 7.17	13.64 ***
Social interaction	6.63 ± 2.19	8.09 ± 2.09	−5.31 ***	7.57 ± 1.80	9.34 ± 1.60	−7.10 ***
Subjected support	18.85 ± 2.54	33.02 ± 112.12	−1.48	25.52 ± 70.02	36.53 ± 121.22	−0.76
Instrumental support	9.85 ± 3.23	11.47 ± 1.78	−4.97 ***	9.07 ± 3.81	11.45 ± 1.22	−5.74 ***
Impulsivity	12.62 ± 6.38	9.69 ± 4.07	4.37 ***	11.16 ± 4.87	9.70 ± 4.65	2.09 *
Mental disorder			80.29 ***			10.686 ***
Yes	57 (52.8)	4 (2.9)		10 (10.9)	0 (0.0)	
No	51 (47.2)	133 (97.1)		82 (89.1)	93 (100.0)	

Note: *: *p* ≤ 0.05; **: *p* ≤ 0.01; ***: *p* ≤ 0.001.

## Data Availability

The datasets used and/or analyzed during the current study are available from the corresponding author on reasonable request.

## References

[1] Suicide worldwide in 2019: Global Health Estimates. https://www.who.int/publications/i/item/9789240026643.

[2] Maris R.W., Berman A.L., Silverman M.M. (2000). Comprehensive Textbook of Suicidology.

[3] Phillips M.R., Li X., Zhang Y. (2002). Suicide rates in China, 1995–99. Lancet.

[4] Zhang J., Sun L., Liu Y., Zhang J. (2014). The Change in Suicide Rates between 2002 and 2011 in China. Suicide Life-Threat. Behav..

[5] Preventing Suicide: A Global Imperative. https://www.who.int/publications/i/item/9789241564779.

[6] Sun J., Guo X., Zhang J., Jia C., Xu A. (2013). Suicide rates in Shandong, China, 1991–2010: Rapid decrease in rural rates and steady increase in male–female ratio. J. Affect. Disord..

[7] Claassen C.A., Harvilchuck-Laurenson J.D., Fawcett J. (2014). Prognostic Models to Detect and Monitor the Near-Term Risk of Suicide: State of the science. Am. J. Prev. Med..

[8] Beck R.W., Morris J.B., Beck A.T. (1974). Cross-Validation of the Suicidal Intent Scale. Psychol. Rep..

[9] Beck A.T. (1991). Beck Scale for Suicide Ideation.

[10] Harriss L., Hawton K., Zahl D. (2005). Value of measuring suicidal intent in the assessment of people attending hospital following self-poisoning or self-injury. Br. J. Psychiatry.

[11] Stefansson J., Nordström P., Jokinen J. (2012). Suicide Intent Scale in the prediction of suicide. J. Affect. Disord..

[12] Clopton J.R., Jones W.C. (1975). Use of the MMPI in the prediction of suicide. J. Clin. Psychol..

[13] Beck A.T., Ward C.H., Mendelson M., Mock J., Erbaugh J. (1961). An inventory for measuring depression. Arch. Gen. Psychiatry.

[14] Beck A.T. (1993). Manual for the Beck Hopelessness Scale.

[15] Carroll B.J. (1982). The Dexamethasone Suppression Test for Melancholia. Br. J. Psychiatry.

[16] López-Ibor J.J.J., Saiz-Ruiz J., Cobos J.P.D.L. (1985). Biological Correlations of Suicide and Aggressivity in Major Depressions (with Melancholia): 5-Hydroxyindoleacetic Acid and Cortisol in Cerebral Spinal Fluid, Dexamethasone Suppression Test and Therapeutic Response to 5-Hydroxytryptophan. Neuropsychobiology.

[17] Ryan C.J., Large M.M., Callaghan S. (2013). Suicide risk assessment: Where are we now?. The Medical Journal of Australia.

[18] mhGAP Intervention Guide for Mental, Neurological and Substance Use Disorders in Non-Specialized Health Settings (Version 2.0). https://www.who.int/publications/i/item/9789241549790.

[19] Posner K., Oquendo M.A., Gould M., Stanley B., Davies M. (2007). Columbia Classification Algorithm of Suicide Assessment (C-CASA): Classification of Suicidal Events in the FDA’s Pediatric Suicidal Risk Analysis of Antidepressants. Am. J. Psychiatry.

[20] Patterson W.M., Dohn H.H., Bird J., Patterson G.A. (1983). Evaluation of suicidal patients: The SAD PERSONS scale. J. Psychosom. Res..

[21] Conrad A.K., Jacoby A.M., Jobes D.A., Lineberry T.W., Shea C.E., Ewing T.D.A., Schmid P.J., Ellenbecker S.M., Lee J.L., Fritsche K. (2009). A Psychometric Investigation of the Suicide Status Form II with a Psychiatric Inpatient Sample. Suicide Life-Threat. Behav..

[22] Dimeff L.A., Jobes D.A., Chalker S.A., Piehl B.M., Duvivier L.L., Lok B.C., Zalake M., Chung J., Koerner K. (2020). A novel engagement of suicidality in the emergency department: Virtual Collaborative Assessment and Management of Suicidality. Gen. Hosp. Psychiatry.

[23] Guo J., Lu Q., Chen X., Liu F. (2018). Retranslation and test of the reliability and validity for suicide risk assessment Scale. Chin. J. Pract. Nurs..

[24] Wang Z., Bian Q., He J., Shu J., Kong Y., Yang L., Zhou J., Chen S. (2019). Reliability and validity analysis of the Columbia Suicide Screening Questionnaire in middle school students. Chin. J. Behav. Med. Brain Sci..

[25] Tan R., Deying H., Liu Y., Ke X., Wang Y., Zhou Y., Teng F. (2018). Development of an indicator system for evaluating mental health in inpatients. Chin. J. Nurs..

[26] Yang X., Tong H. (2010). Suicidal Risk Assessment and Related Social-psychological Factors in College Students. Chin. J. Clin. Psychol..

[27] Posner K., Brown G.K., Stanley B., Brent D.A., Yershova K.V., Oquendo M.A., Currier G.W., Melvin G., Greenhill L., Shen S. (2011). The Columbia–Suicide Severity Rating Scale: Initial Validity and Internal Consistency Findings from Three Multisite Studies with Adolescents and Adults. Am. J. Psychiatry.

[28] Jobes D.A., Kahn-Greene E., Greene J.A., Goeke-Morey M. (2009). Clinical Improvements of Suicidal Outpatients: Examining Suicide Status Form Responses as Predictors and Moderators. Arch. Suicide Res..

[29] Jobes D.A. (2012). The Collaborative Assessment and Management of Suicidality (CAMS): An Evolving Evidence-Based Clinical Approach to Suicidal Risk. Suicide Life-Threat. Behav..

[30] Wang Z., Xie B., Bian Q., Wan L. (2015). Reliability and validity of the Columbia Suicide Screen for suicide risk assessment in senior middle school students. Chin. J. Psychiatry.

[31] Zhao F. (2011). Comprehensive evaluation of agricultural development scale among the provinces and cities in China. China Secur. Futures.

[32] Fang L., Zhang J. (2010). Validity of Proxy Data Obtained by Different Psychological Autopsy Information Reconstruction Techniques. J. Int. Med. Res..

[33] Zhang J., Wieczorek W., Conwell Y., Tu X.-M., Wu B.Y.-W., Xiao S., Jia C. (2009). Characteristics of young rural Chinese suicides: A psychological autopsy study. Psychol. Med..

[34] Zhang J., Li Z.-Y., Xiao S.-Y., Zhou L., Jia C.-X., Pan G.-W. (2012). Mental disorder and suicide among youths in rural China: A case control study based on consecutive samples from Hunan, Liaoning and Shandong provinces. Chin. J. Epidemiol..

[35] Sun L., Zhang J. (2017). Gender differences among medically serious suicide attempters aged 15–54 years in rural China. Psychiatry Res..

[36] Zhang J., Lin L. (2013). The Moderating Effects of Impulsivity on Chinese Rural Young Suicide. J. Clin. Psychol..

[37] Zhang J., Xu H. (2007). The Effects of Religion, Superstition, and Perceived Gender Inequality on the Degree of Suicide Intent: A Study of Serious Attempters in China. Omega J. Death Dying.

[38] Lorant V., Kunst A.E., Huisman M., Costa G., Mackenbach J. (2005). Socio-economic inequalities in suicide: A European comparative study. Br. J. Psychiatry.

[39] Jia C.-X., Zhang J. (2011). Characteristics of Young Suicides by Violent Methods in Rural China. J. Forensic Sci..

[40] Paykel E.S., Prusoff B.A., Uhlenhuth E.H. (1971). Scaling of Life Events. Arch. Gen. Psychiatry.

[41] Zhang J., Ma Z. (2012). Patterns of life events preceding the suicide in rural young Chinese: A case control study. J. Affect. Disord..

[42] Kong Y.Y., Zhang J., Jia S.H., Zhou L. (2007). Reliability and Validity of the Beck Hopelessness Scale for Adolescents. Chin. Ment. Health J..

[43] Koenig H.G., Westlund R.E., George L.K., Hughes D.C., Blazer D.G., Hybels C. (1993). Abbreviating the Duke Social Support Index for Use in Chronically Ill Elderly Individuals. J. Psychosom. Res..

[44] Jia C., Zhang J. (2012). Psychometric characteristics of the Duke Social Support Index in a young rural Chinese population. Death Stud..

[45] Dickman S.J. (1990). Functional and dysfunctional impulsivity: Personality and cognitive correlates. J. Pers. Soc. Psychol..

[46] Gao Q., Zhang J., Jia C. (2011). Psychometric properties of the Dickman Impulsivity Instrument in suicide victims and living controls of rural China. J. Affect. Disord..

[47] First M.B., Spitzer R.L., Gibbon M., Williams J.B.W. (2002). Structured Clinical Interview for DSM-IV-TR Axis I Disorders, Research Version, Patient Edition. (SCID-I/P).

[48] Sun L., Zhang J. (2014). Characteristics of Chinese rural young suicides: Who did not have a strong intent to die. Compr. Psychiatry.

[49] Sun L., Zhang J. (2014). Coping Skill as a Moderator Between Negative Life Events and Suicide Among Young People in Rural China. J. Clin. Psychol..

[50] Zhang J., Conwell Y., Zhou L., Jiang C. (2004). Culture, risk factors and suicide in rural China: A psychological autopsy case control study. Acta Psychiatr. Scand..

[51] Zhang J., Li N., Tu X.-M., Xiao S., Jia C. (2011). Risk factors for rural young suicide in China: A case–control study. J. Affect. Disord..

[52] Lyu J., Wang Y., Shi H., Zhang J. (2018). Early warnings for suicide attempt among Chinese rural population. J. Affect. Disord..

[53] Beck A.T., Steer R.A., Kovacs M., Garrison B. (1985). Hopelessness and eventual suicide: A 10-year prospective study of pa-tients hospitalized with suicidal ideation. Am. J. Psychiatry.

[54] Bolton J.M., Robinson J. (2010). Population-Attributable Fractions of Axis I and Axis II Mental Disorders for Suicide Attempts: Findings from a Representative Sample of the Adult, Noninstitutionalized US Population. Am. J. Public Health.

[55] Bagge C.L., Glenn C.R., Lee H.-J. (2013). Quantifying the impact of recent negative life events on suicide attempts. J. Abnorm. Psychol..

[56] Assink M., Van Der Put C.E., Stams G.J.J.M. (2014). The Development and Validation of an Actuarial Risk Assessment Tool for the Prediction of First-Time Offending. Int. J. Offender Ther. Comp. Criminol..

[57] Simon G.E., Johnson E., Lawrence J.M., Rossom R.C., Ahmedani B., Lynch F.L., Beck A., Waitzfelder B., Ziebell R., Penfold R.B. (2018). Predicting Suicide Attempts and Suicide Deaths Following Outpatient Visits Using Electronic Health Records. Am. J. Psychiatry.

[58] Hawes M., Yaseen Z., Briggs J., Galynker I. (2016). The Modular Assessment of Risk for Imminent Suicide (MARIS): A proof of concept for a multi-informant tool for evaluation of short-term suicide risk. Compr. Psychiatry.

[59] Qin P., Mortensen P.B. (2001). Specific characteristics of suicide in China. Acta Psychiatr. Scand..

[60] Eskin M. (2004). The effects of religious versus secular education on suicide ideation and suicidal attitudes in adolescents in Turkey. Soc. Psychiatry Psychiatr. Epidemiology.

[61] Zhang J., Thomas D.L., Judd D.K. (1999). Familial and Religious Influences on Suicidal Ideation. Religion, Mental Health and the Latter-Day Saints.

[62] Wortzel H.S., Nazem S., Bahraini N.H., Matarazzo B.B. (2017). Why Suicide Risk Assessment Still Matters. J. Psychiatr. Pract..

